# Nitrogen Enhances Salt Tolerance by Modulating the Antioxidant Defense System and Osmoregulation Substance Content in *Gossypium hirsutum*

**DOI:** 10.3390/plants9040450

**Published:** 2020-04-03

**Authors:** Ripon Kumar Sikder, Xiangru Wang, Hengheng Zhang, Huiping Gui, Qiang Dong, Dingsha Jin, Meizhen Song

**Affiliations:** 1 State Key Laboratory of Cotton Biology, Institute of Cotton Research, Chinese Academy of Agricultural Sciences, Anyang 455000, Henan, China; 2017Y90100144@caas.cn (R.K.S.); wangxiangru@caas.cn (X.W.); zhanghengheng@caas.cn (H.Z.); guihuiping@caas.cn (H.G.); dongqiang@caas.cn (Q.D.); xuyc@cricaas.com.cn (D.J.); 2 School of Agricultural Sciences, Zhengzhou University, Zhengzhou 450001, China

**Keywords:** cotton, NaCl stress, nitrogen, antioxidant defense system, osmotic adjustment

## Abstract

Increasing soil salinity suppresses both productivity and fiber quality of cotton, thus, an appropriate management approach needs to be developed to lessen the detrimental effect of salinity stress. This study assessed two cotton genotypes with different salt sensitivities to investigate the possible role of nitrogen supplementation at the seedling stage. Salt stress induced by sodium chloride (NaCl, 200 mmol·L^−1^) decreased the growth traits and dry mass production of both genotypes. Nitrogen supplementation increased the plant water status, photosynthetic pigment synthesis, and gas exchange attributes. Addition of nitrogen to the saline media significantly decreased the generation of lethal oxidative stress biomarkers such as hydrogen peroxide, lipid peroxidation, and electrolyte leakage ratio. The activity of the antioxidant defense system was upregulated in both saline and non-saline growth media as a result of nitrogen application. Furthermore, nitrogen supplementation enhanced the accumulation of osmolytes, such as soluble sugars, soluble proteins, and free amino acids. This established the beneficial role of nitrogen by retaining additional osmolality to uphold the relative water content and protect the photosynthetic apparatus, particularly in the salt-sensitive genotype. In summary, nitrogen application may represent a potential strategy to overcome the salinity-mediated impairment of cotton to some extent.

## 1. Introduction

Salt stress is the foremost environmental factor that drastically decreases the crop yield in different geographic areas, especially in arid and semi-arid regions [1,2]. This has been forecast to gradually increase as a consequence of climate change [3]. About 7% of the total global land area is affected by the detrimental effects of salinity [4]. It has also been projected that 20% of the total cultivable land and 33% of the irrigated agricultural land are affected by salt, an area that increases at an alarming rate of 10% annually, equating to a 2.0% and 3.3% increase per annum, respectively [5,6]. In China, approximately 4.88% of the total available land is affected by salinity [7].

Cotton (*Gossypium hirsutum* L.) is considered a moderately salt-tolerant and non-halophytic plant that may also be used as a pioneer crop for soil reclamation of saline-alkaline land. However, the growth and productivity of cotton are adversely affected by high salinity levels and their tolerance level depends on the genetic background of plants and their developmental stage [8]. Usually, cotton plants exhibited most sensitivity to salinity during the seedling growth stage, and remained sensitive at the reproductive stage [9]. Excess salinity inhibits plant growth and development by inducing osmotic stress, particular ion toxicity (Na^+^ and Cl^−^), oxidative damage, and/or nutritional disorders in plant tissues, which subsequently lead to weakened plant growth and endurance [10,11,12]. Salt stress has been shown to significantly decrease the uptake and metabolism of numerous mineral nutrients (such as nitrogen, potassium, phosphorus, and calcium), and accelerate the damage of the photosynthetic machinery as well as the whole salt tolerance mechanisms of plants. Salt stress-induced plant metabolism alterations are the secondary consequence of carbon and nitrogen metabolism [13]. Moreover, a high salt concentration perturbs electron transport systems in both chloroplasts and mitochondria [14,15]. This accelerates electron leakage from electron transportation chains and induces the generation of reactive oxygen species (ROS), such as superoxide radicals, hydroxyl radicals, and hydrogen peroxide [16,17]. The over-accumulation of ROS not only damages cellular and biological activities [18,19,20], but also degrades chlorophyll pigments and obstructs the synthesis of proteins, amino acids, lipids, deoxyribonucleic acid, as well as enzymatic and non-enzymatic activities of plants [17]. To avoid ROS-induced oxidative damage, plant-assured endogenous tolerance approaches, such as the antioxidant defense system as well as the accumulation of organic solutes and secondary metabolites [21]. Superoxide dismutase (SOD), catalase (CAT), and peroxidase (POD) are considered active oxygen-scavenging enzymes. SOD scavenges toxic singlet oxygen and consequently converts it to hydrogen peroxide under the active participation of POD. CAT decomposes hydrogen peroxide and superoxide radicals while POD degrades hydrogen peroxide by oxidation of various phenolic compounds [19]. Apart from antioxidants, the plant also accumulates various osmolytes, such as sugar, proteins, amino acids, and glycine betaine, for metabolic modification to overcome salt-induced lethal effects. Organic osmolytes are deposited within vacuoles of the cytoplasm to maintain osmotic adjustment, protect cell membranes, and denature protein by retaining the cell turgor [6,22]. It has been reported that plants with an improved antioxidant defense system, stronger metabolism of organic osmolytes, and higher accumulation of essential mineral nutrients display better salt tolerance [23,24].

The adaptation of plants to salt stress may result in several aspects that function at the ionic, morphological, physiological, biochemical, and genetic levels [25,26]. Diverse approaches have been taken to decrease the negative impact of salt stress on plants such as the cultivation of new salt tolerant varieties and measures to improve soil salinization. However, these measures require a very long time to implement. Previous studies indicated that proper mineral nutrition or supplementation with plant-growth regulators may be one of the best-suited strategies to improve the tolerance potential of a plant [8,19,27]. Nitrogen is not only a vital macronutrient for cotton production [14,28,29], but also the next crucial input factor for better crop production after water [30]. Moreover, nitrogen governs many cellular activities in plants since it is part of key components of chlorophyll, amino acids, proteins, nucleic acids, enzymes, plant hormones, and osmolytes [17,27], all of which are involved in plant salt tolerance mechanisms through different pathways [31,32]. NO_3_^−^ and NH_4_^+^ are the most common inorganic forms of nitrogen, and plants usually uptake these by their roots and use them for their metabolic activities [33]. Therefore, this study speculated that the additional application of nitrogen will enhance the salt tolerance of cotton. In fact, recent studies have reported that exogenous nitrogen application significantly modulated the growth of tomato plants [17], *Brassica* genotypes [34], and wheat seedlings [21] during exposure to salt stress. This modulation is achieved by enhancing photosynthetic performance, upregulating antioxidant activity, and reducing oxidative stress. However, the interaction of nitrogen with NaCl in cotton remains poorly characterized and, furthermore, little has been documented on the interaction of nitrogen and salt in other crop species. Therefore, this study was conducted to investigate the role of nitrogen with the goal to enhance the salt tolerance of cotton genotypes. This study hypothesized that (a) nitrogen could lessen the salinity stress of cotton seedlings by upregulating their antioxidant defense system, while maintaining the redox potential, and; (b) the effect of nitrogen on the salt tolerance level may vary noticeably between cotton genotypes with different salt tolerances.

## 2. Results

### 2.1. Nitrogen Supplementation Increased Growth and Biomass Accumulation

**Figure 1:**
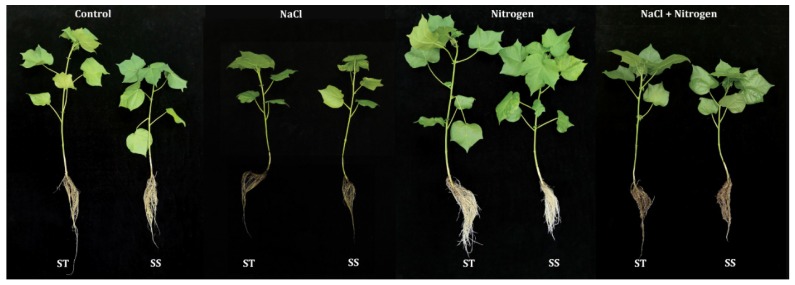
Morphological representation of cotton genotypes under different treatments.

**Figure 2:**
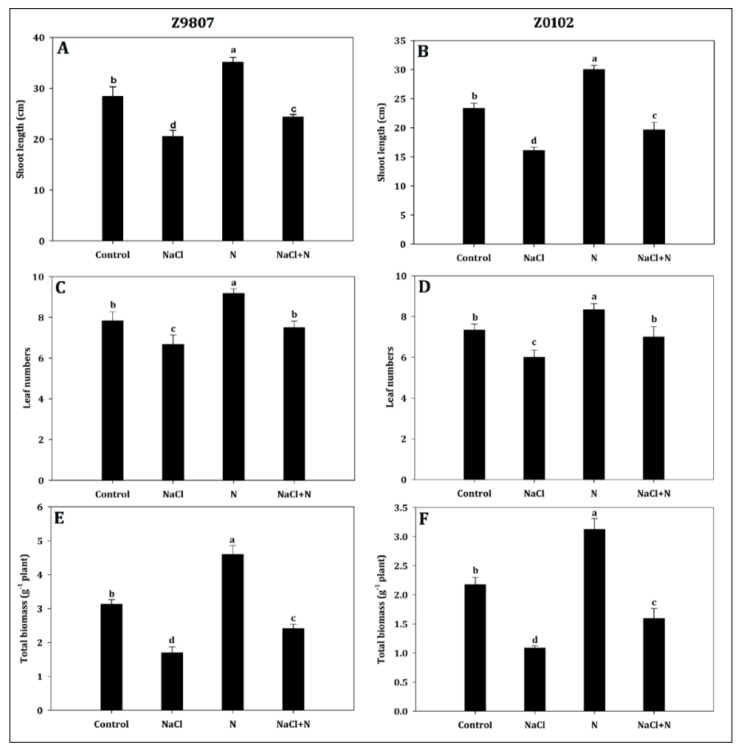
Effect of nitrogen supplementation on (**A**,**B**) shoot length, (**C**,**D**) leaf numbers, and (**E**,**F**) total biomass accumulation in *Gossypium hirsutum* L. under salinity stress. Data represent the mean of three replicates and bars with different letters denote significant differences at *p* < 0.05.

Cotton seedlings grown in the saline environment showed noticeably decreased growth parameters including shoot length (SL), leaf number (LN) per plant, and total biomass production compared with the non-saline environment (Figure 1 and Figure 2). Moderate nitrogen application to salt-stressed seedlings noticeably alleviated the salinity damage by progressively increasing SL, LN, and total biomass of salt tolerant (ST) (by 18.6%, 12.5%, and 42.3%) and salt sensitive (SS) (by 22.3%, 16.7%, and 46.7%) genotypes compared to their salt-stressed counterparts (Figure 2A–F). However, seedling growth under saline conditions was still inhibited compared with the corresponding non-saline conditions even though the availability of nitrogen exerted a positive effect.

### 2.2. Nitrogen Addition Improves Pigment Synthesis

**Figure 3:**
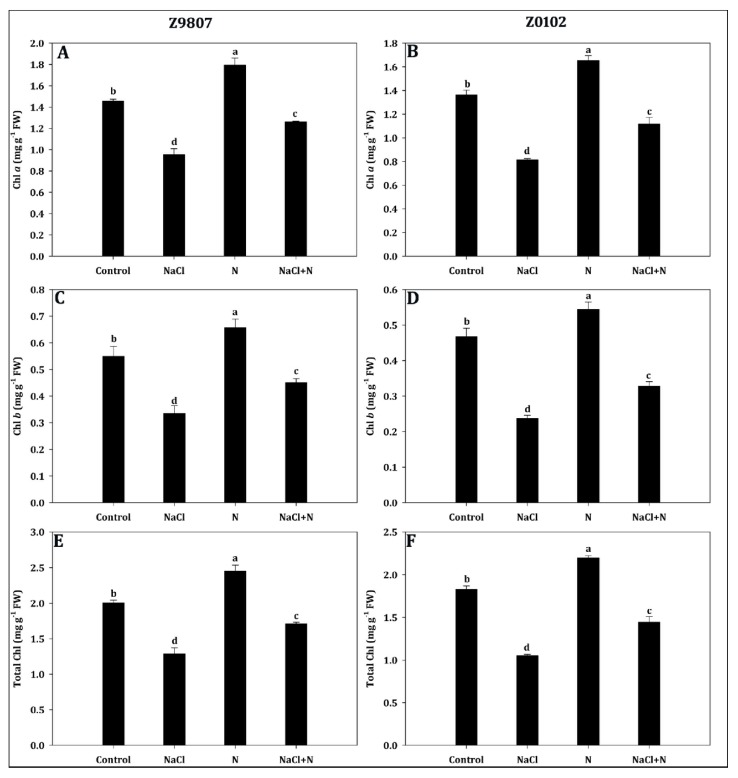
Effect of nitrogen supplementation on (**A**,**B**) chlorophyll *a*, (**C**,**D**) chlorophyll *b*, and (**E**,**F**) total chlorophyll content in *Gossypium hirsutum* L. under salinity stress. Data represent the mean of three replicates and bars with different letters denote significant differences at *p* < 0.05.

The results showed that nitrogen supplementation significantly enhanced the chlorophyll *a* (Chl *a*), chlorophyll *b* (Chl *b*), and total chlorophyll (TChl) contents of cotton seedlings (Figure 3). Relative to control, 2.5 mmol·L^−1^ nitrogen increased the Chl *a* content by 23.2%, the Chl *b* content by 19.6%, and the TChl content by 22.2% in the ST genotype whereas in the SS genotype, the values increased by 21.2%, 16.5%, and 20.0%, respectively. Salt stress decreased the Chl *a*, Chl *b*, and TChl contents by 34.5%, 39.1%, and 35.8% in the ST genotype and by 40.4%, 49.3%, and 42.6% in the SS genotype, respectively, compared with the control condition. However, nitrogen application to salt-stressed plants led to apparent increases of 32.7% for Chl *a*, 34.4% for Chl *b*, and 32.8% for TChl in the ST genotype while in the SS genotype, these increased by 37.3%, 38.5%, and 37.5%, respectively (Figure 3A–F). This showed that nitrogen fertilization promoted chlorophyll synthesis under salt stress, especially in the SS genotype.

### 2.3. Influence of Nitrogen on Gas Exchange Attributes in Response to Salinity Stress

**Figure 4:**
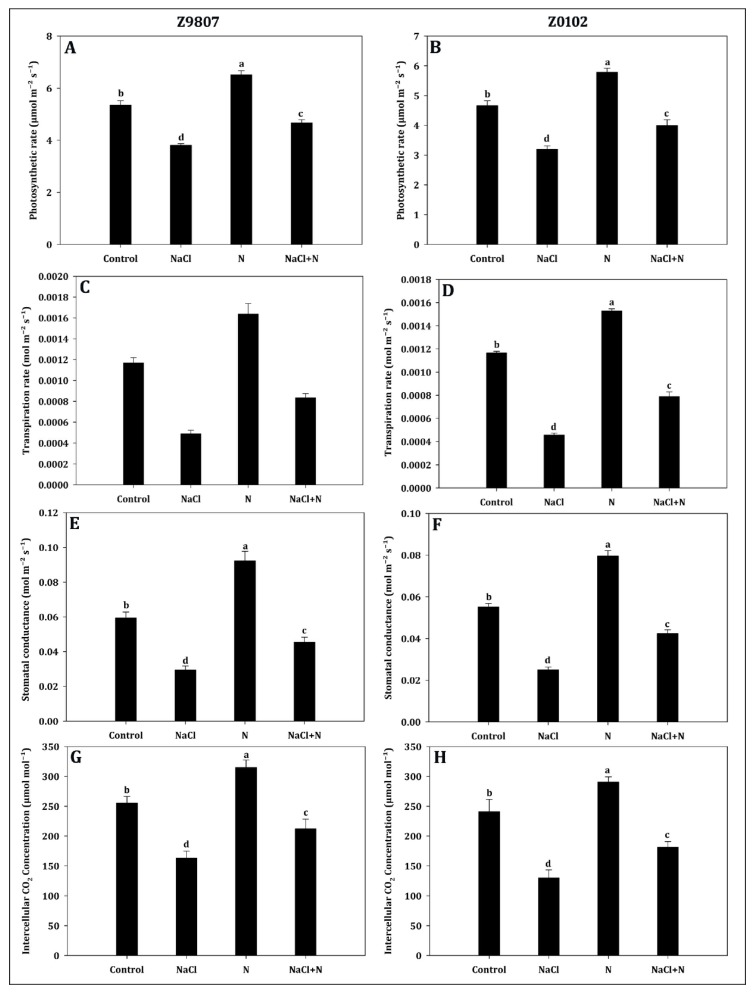
Effect of nitrogen supplementation on (**A**,**B**) photosynthetic rate, (**C**,**D**) transpiration rate and (**E**,**F**) stomatal conductance, and (**G**,**H**) intercellular CO_2_ concentration and in *Gossypium hirsutum* L. under salinity stress. Data represent the mean of three replicates and bars with different letters denote significant differences at *p* < 0.05.

The data associated with the gas exchange attributes is shown in Figure 4. The results showed that salt stress noticeably affected the photosynthetic rate (A), transpiration rate (E), stomatal conductance (gsw), and intercellular CO_2_ concentration (Ci) compared with the untreated control environment of both genotypes. The seedlings subjected to 2.5 mmol·L^−1^ nitrogen treatment alone had significantly increased gas exchange parameters compared with the control. Furthermore, nitrogen supplementation in the salt-stressed seedlings amends this detrimental effect of NaCl. A, E, gsw, and Ci increased by 22.6%, 71.3%, 54.1%, and 30.2% in ST genotype and 25.0%, 73.3%, 69.5%, and 39.5% in SS genotype, respectively, compared with salt-treated seedlings alone (Figure 4A–H).

### 2.4. Nitrogen Induces Osmolyte Accumulation under Salt Stress

**Figure 5:**
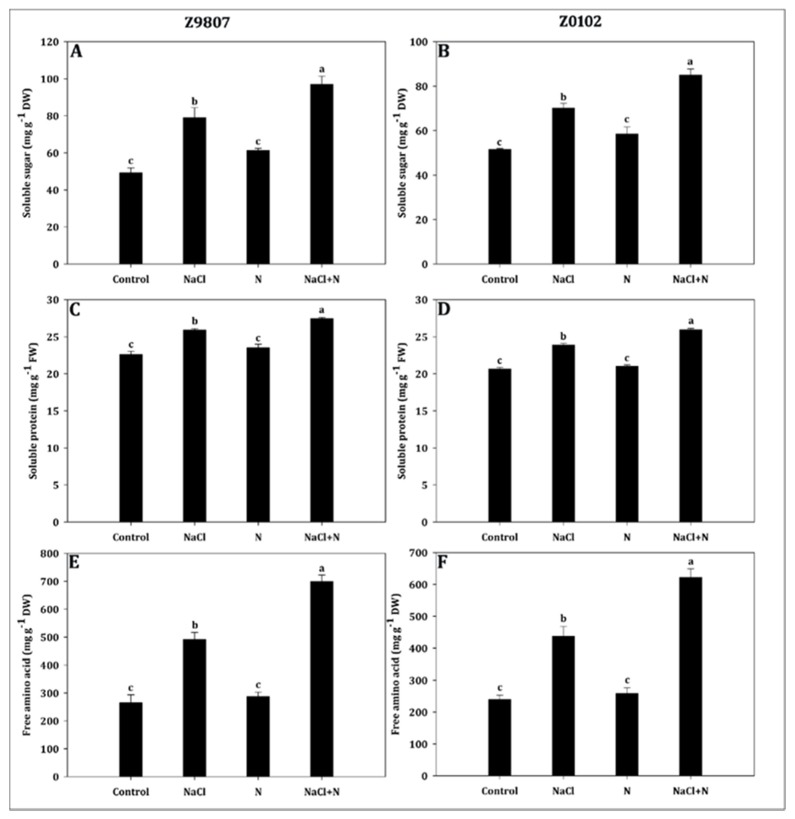
Effect of nitrogen supplementation on (**A**,**B**) soluble sugar, (**C**,**D**) soluble protein, and (**E**,**F**) free amino acid in *Gossypium hirsutum* L. under salinity stress. Data represent the mean of three replicates and bars with different letters denote significant differences at *p* < 0.05.

Nitrogen availability progressively increased the accumulation of soluble sugar, soluble protein, and free amino acids and the maximum accumulation was observed in nitrogen only supplemented seedlings (Figure 5). Compared with untreated control seedlings, soluble sugar, soluble protein, and free amino acid contents increased in the ST genotype by 24.4%, 4.0%, and 8.5% as well as by 13.3%, 1.7%, and 8.0% in the SS genotype, respectively, as a result of nitrogen supplementation. The salt-stressed seedlings supplied with high nitrogen accumulated more osmotic-adjustment substances compared with salt-stressed treatment alone. For ST and SS genotypes, the most significant increases were 22.6% and 21.4% for soluble sugar, 5.9% and 8.5% for soluble protein, and 42.2% and 42.2% for free amino acid, respectively (Figure 5A–F).

### 2.5. Application of Nitrogen Improves the Plant Water Status

**Figure 6:**
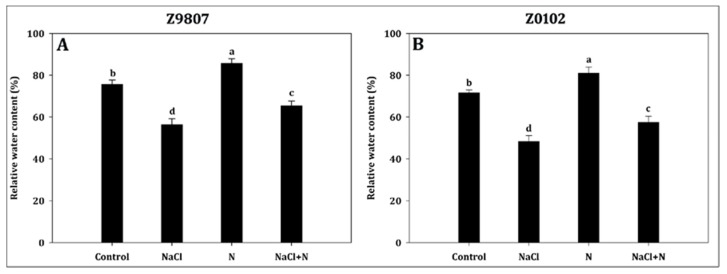
Effect of nitrogen supplementation on leaf relative water content in *Gossypium hirsutum* L. under salinity stress. Data represent the mean of three replicates and bars with different letters denote significant differences at *p* < 0.05.

Nitrogen addition to the growth media increased the leaf relative water content (LRWC) as shown in Figure 6. In response to salt stress, the LRWC decreased significantly by 25.6% and 32.6% in ST and SS genotypes, respectively, compared with untreated control seedlings. Nitrogen supplementation in salt-stressed (NaCl + N) seedlings mitigated the toxic effect of NaCl by gradually increasing the LRWC by 16.3% in ST genotype and by 19.2% in the SS genotype compared with salt-stressed seedlings (Figure 6A,B).

### 2.6. Application of Nitrogen Mitigates Oxidative Damage

**Figure 7:**
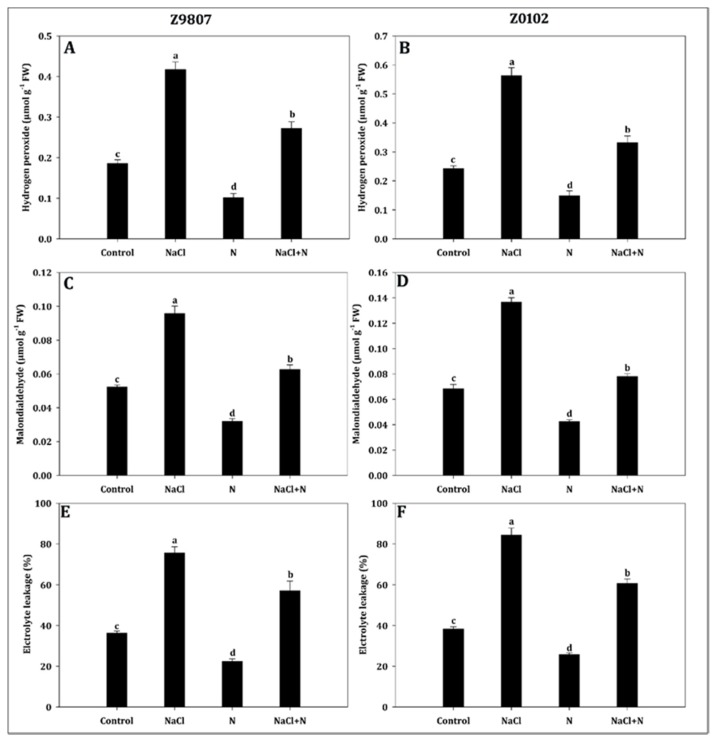
Effect of nitrogen supplementation on (**A**,**B**) hydrogen peroxide, (**C**,**D**) malondialdehyde, and (**E**,**F**) electrolyte leakage ratio in *Gossypium hirsutum* L. under salinity stress. Data represent the mean of three replicates and bars with different letters denote significant differences at *p* < 0.05.

Nitrogen significantly alleviated the damaging effect of hydrogen peroxide and, therefore, averting lipid peroxidation (MDA content) and electrolyte leakage (EL) ratio (Figure 7). Salt stress increased the hydrogen peroxide content by 2.2 and 2.3 times in ST and SS genotypes, respectively, compared with untreated control seedlings (Figure 7A,B). This caused gradual increases in MDA and EL by 1.8 and 2.1 times in the ST genotype and by 2.0 and 2.2 times in the SS genotype, respectively (Figure 7C–F). Compared with untreated control seedlings, nitrogen supplementation decreased hydrogen peroxide generation, and caused a decrease of lipid peroxidation and electrolyte leakage ratio in both genotypes. Moreover, nitrogen addition to salt-stressed seedlings caused maximal alleviation of hydrogen peroxide generation (1.5 times in ST and 1.7 times in SS), which resulted in a decrease of MDA (1.5 times in ST and 1.7 times in SS) and electrolyte leakage (1.3 times in ST and 1.4 times in SS). This contrasts with the salt-stressed treatment alone.

### 2.7. Nitrogen Upregulates the Antioxidant Activity of Cotton Genotypes under Salinity Stress

**Figure 8:**
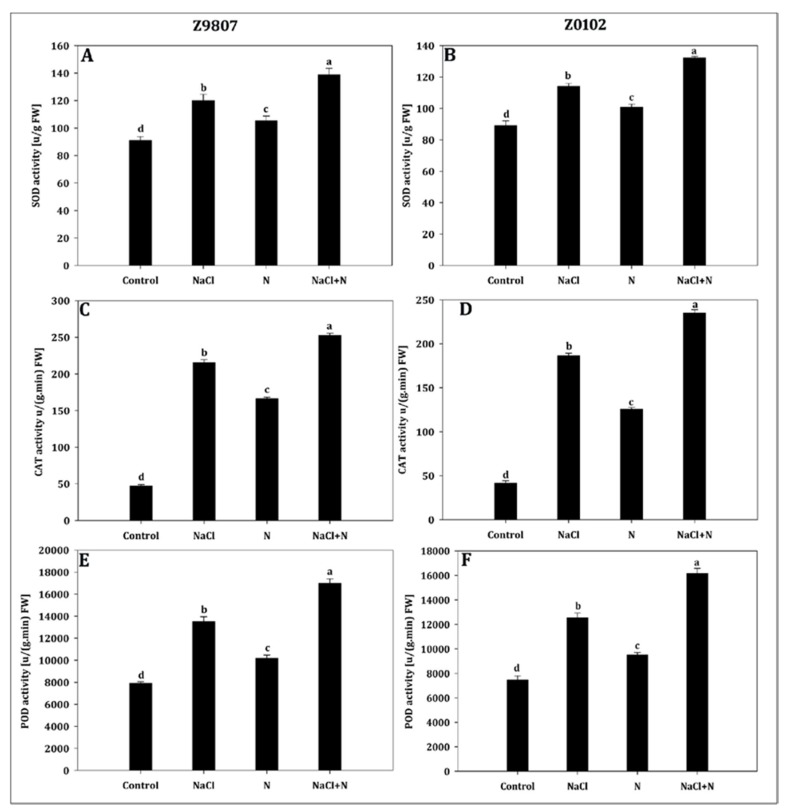
Effect of nitrogen supplementation on (**A**,**B**) superoxide dismutase, (**C**,**D**) catalase, and (**E**,**F**) peroxidase in *Gossypium hirsutum* L. under salinity stress. Data represent the mean of three replicates and bars with different letters denote significant differences at *p* < 0.05.

Nitrogen availability in the growth media markedly increased the antioxidant activities in cotton seedlings by upregulating SOD, CAT, and POD activities (Figure 8). Compared with the untreated control, nitrogen only enhanced SOD, CAT, and POD activity by 1.2-, 3.5-, and 1.3-fold in the ST genotype and by 1.1-, 3.0-, and 1.3-fold in the SS genotype, respectively. In salt-stressed cotton seedlings, SOD, CAT, and POD activities increased by 1.3-, 4.6-, and 1.7-fold in the ST genotype and by 1.3-, 4.4-, and 1.7-fold in the SS genotype, respectively, compared with the untreated control. Upon nitrogen supplementation in NaCl-stressed seedlings, increases of SOD (1.16-fold in ST and SS), CAT (1.17-fold in ST and 1.26-fold in SS), and POD (1.26-fold in ST and 1.29-fold in SS) were observed compared with salt-treated seedlings alone (Figure 8A–F).

### 2.8. Cluster Heat Map Analysis

**Figure 9:**
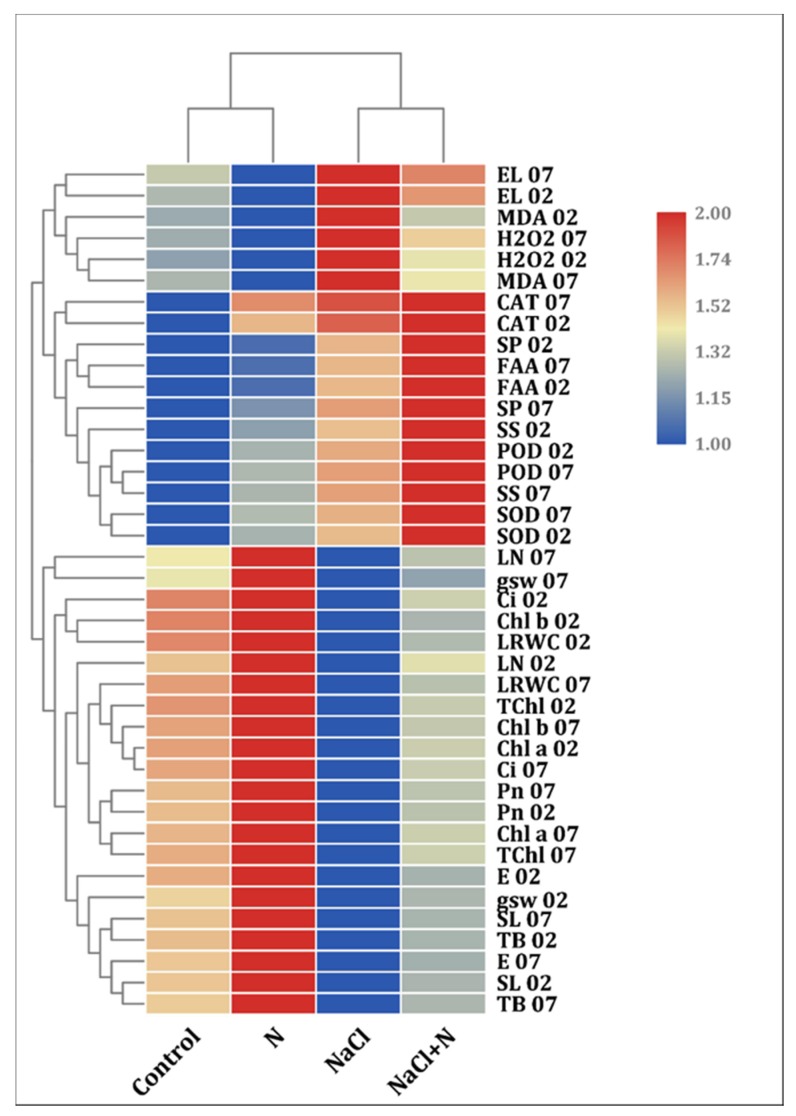
Heat map showing different morpho-physiological traits of cotton genotypes according to different treatments. Measured traits are arranged in rows and columns represents different treatments.

Heat map analysis clearly identified the overall variations among all four treatments (Figure 9). Nitrogen treatment alone significantly increased all studied traits compared with the non-salt-stressed control seedlings in both ST and SS genotypes except for oxidative stress biomarkers. However, nitrogen supplementation on the stressed seedlings exerted a positive role through decreasing oxidative injury by enhancing photosynthetic performance, increasing the antioxidant metabolism, and accumulating more osmolytes as well as significantly improving the plant water status.

## 3. Discussion

The seedling stage is a crucial and particularly vulnerable stage in the life cycle of crops and healthy and strong seedlings guarantee a considerable yield in a population. As a major abiotic stress worldwide, salt at low concentration suppresses plant growth and productivity while high salinity can cause the death of plants [4,35,36,37]. Hussain et al. [37] reported that the root length, root number, and shoot length of rice seedlings were significantly decreased in response to salt stress, and higher salt concentrations caused higher leaf mortality in all rice cultivars at the early seedling stage [38]. Singh et al. [17] reported a significant loss in growth (such as short plant height, lower biomass, and less photosynthetic pigments) and gross photosynthesis in tomato seedlings treated with salt. Inhibition of root and shoot growth, leaf photosynthesis rate, maximum photochemistry efficiency of photosystem II, and chlorophyll were observed in wheat and cotton seedlings that were exposed to saline conditions [39,40]. Similar to the previous studies mentioned above, this study demonstrated significant adverse effects of salinity on cotton seedling growth and the resulting physiological characteristics under hydroponic culture. Specifically, salt stress decreased the SL, NL per plant, biomass (Figure 1), photosynthetic pigment synthesis, and gas exchange attributes in both tested genotypes (Figure 2, Figure 3 and Figure 4). Moreover, the higher reduction in the SS cultivar indicated a more severe adverse effect of salinity on the SS cultivar, which is consistent with previous reports [41,42]. Recent studies in tomato [17], wheat [21], *Hordeum vulgare* [43], *Carrizo citrange* [44], *Spartina alterniflora* [45], *Sorghum bicolor* [46], and *Brassica* [34] showed that higher nitrogen supplementation can regulate the salinity tolerance and improve plant growth. In the experiments reported here, application of 2.5 mmol·L^−1^ nitrogen increased the SL, NL per plant, and total biomass in both ST and SS genotypes under salt stress (Figure 2). These results indicate that the nitrogen availability not only plays an important role under unstressed conditions but also increased the salt tolerance under salt-stressed conditions, especially for the SS cotton genotype.

It is well known that the accumulation of ROS is central to the plant response to stress [47]. Excessive ROS causes a series of damages, such as the loss of protease activity, membrane lipid peroxidation, and respiratory chain disorder [48]. MDA is commonly used as a biomarker to measure the oxidative damage of the plasmalemma and intracellular organelles in response to abiotic stresses such as high salinity [49]. After 14 days of 200 mmol·L^−1^ NaCl treatment, the MDA content increased sharply in the leaves of seedlings compared with control (Figure 7C,D). Furthermore, the EL of salt-stressed seedlings was higher than that of the control (Figure 7E,F). The significant decrease of the MDA content and EL ratio in the higher nitrogen treatment suggested that nitrogen can protect membranes from salinity-induced damage. ROS scavenging is important for the defense against oxidative damage during stressed conditions [50,51]. In the long-term evolution of plants, two types of protective systems, antioxidant enzymes such as SOD, POD, CAT, ascorbate peroxidase, and glutathione reductase, and non-enzymes such as proline, glutathione, ascorbic acid, and amino acids, have developed. Both endow plants with the ability to scavenge ROS and either decrease or avoid the damage ROS impose on cells [52,53]. Considerable research indicates that salt-tolerant genotypes or species have higher capability for survival or are less affected by salinity. This has partly been attributed to higher activities of their antioxidant enzyme system and/or a higher content of non-enzymatic antioxidants [8,54,55]. Since nitrogen can improve the antioxidant capacity and salt tolerance [21], this study examined the contents of H_2_O_2_, SOD, CAT, POD, and free amino acids. The results showed that the generation rate of H_2_O_2_ was increased by salinity compared with control, especially in the SS genotype. Application of nitrogen effectively inhibited the accumulation of H_2_O_2_ in both genotypes (Figure 7A,B). The elimination of H_2_O_2_ might be due to the immediate strengthening of SOD, CAT, and POD activities (Figure 8), and the increased content of free amino acids (Figure 5E,F) by higher nitrogen application. Moreover, in support of the outcomes of the present study, several other scholars reported that co-application of nitrogen significantly boosted the level of antioxidant enzymes in soybean [56], *Catharanthus roseus* [57], and blueberry [58]. Nitrogen serves as a first line of defense against internal and environmental oxidative stressors [17,21,59,60]. Moreover, the positive effect of nitrogen may be related to nitrogen being an effective component of antioxidant enzymes and antioxidants [17,27].

One of the side-effects of salt stress is osmotic stress. The LRWC decreased significantly under salt treatment, indicating that osmotic stress occurred in the tested cotton seedlings (Figure 6). Osmotic stress caused by soil salinization leads to “physiological drought” in plants, which decreases the water absorption capacity of roots, decreases the internal water supply, and slows down or inhibits water metabolism, transportation, cooling, and other functions that affect plant growth. Lower relative water content induced by salt stress causes stomatal closure and lowers transpiration rate, and, as a result, decreased both CO_2_ and H_2_O intake, and hence decreases photosynthesis [61,62]. Consistent with previous studies, the results of this study showed that cotton seedlings grown under salt stress had decreased growth and pigment synthesis. This resulted in a significant decrease of A as well as other gas exchange attributes (Figure 3 and Figure 4). Mahmood et al. [63] and Jan et al. [64] also reported a salt-induced decrease in the gas exchange attributes in different plant species. Photosynthesis plays a critical role in plant growth since it produces structural carbohydrates (such as lignin and cellulose) that are mainly used in plant morphogenesis. Photosynthesis also produces nonstructural carbohydrates (such as glucose, fructose, sucrose, fructan, and starch) involved in plant life metabolism [65]. Water stress not only affects the carbohydrate distribution, but also the size of the sucrose pool [65,66]. Although the total amount of carbon assimilation decreases in response to drought stress, the content of soluble carbohydrates increases, which can increase the osmotic potential of cells, which corresponds to an increase of sucrose phosphate synthetase activity and sucrose synthesis [65]. Similarly, in this study, the photosynthesis rate sharply decreased, while the soluble sugar content significantly increased in cotton seedlings (Figure 4 and Figure 5). The accumulation of soluble sugar is not only part of the plant metabolism as an osmoprotectant, but also acts as a signaling molecule in the sugar-sensing and signaling system, which helps cotton seedlings to maintain their growth [67].

**Figure 10:**
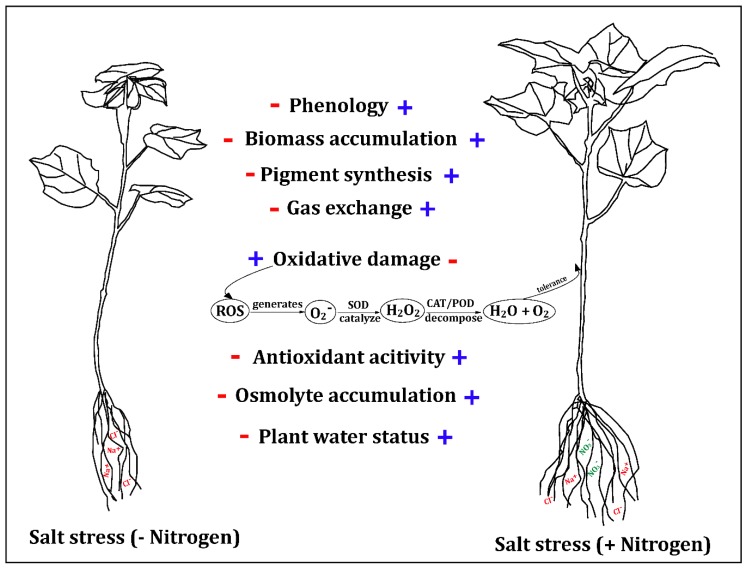
A proposed model for nitrogen interaction with salt stress in cotton seedlings. Salt stress negatively correlated with growth and development by reduces photosynthetic attributes and over production of reactive oxygen species (ROS). Nitrogen supplementation progressively detoxifies the deleterious effect of ROS by stimulating the antioxidant defense system, maintaining better redox homeostasis.

Osmotic regulation is an effective measure for plants to adapt to osmotic stress and avoid dehydration. Organic solutes or osmoprotectants aid plant survival under extreme stress via osmotic adjustment by stabilizing specific proteins and membranes and by preventing dehydration within the organelles of cells [68,69,70]. To adapt to salt stress, most of the glycophytes, including cotton, accumulate a large number of organic solutes in their tissues [71]. In the present study, salinity stress triggered the accumulation of compatible solutes such as soluble sugar, free amino acids, and soluble protein compared with non-saline and salt-stressed plants (Figure 5). A similar accumulation trend of soluble sugar and protein under salt stress was reported by Ahmad et al. [72], Abdel Latef et al. [73], and Liu et al. [74] for chickpeas, Lupine, and *Nitraria tangutorum*, respectively. Total free amino acid also started to escalate under salinity stress in wheat [75] and *Lupinus termis* [73]. In addition, the accumulation of osmoregulators in the ST genotype was higher than that in Z0102, which increased their water absorption ability and salt tolerance (Figure 5). Furthermore, application of nitrogen significantly increased the osmoregulation substance, indicating a significant improvement in osmotic regulation (Figure 5 and Figure 10). The enhancement of osmotic regulation because of the sufficient supply of nitrogen fertilizer ensured the uptake of water and minerals required for the carbon and nitrogen metabolisms. This ultimately ensured a relatively normal plant growth under salinity conditions.

Overall, this study showed that while cotton seedling growth decreased (SL, NL, biomass accumulation, and photosynthesis) under salinity, the availability of effective nitrogen fertilizer alleviated the damage caused by salinity, particularly in the SS genotype, by enhancing both antioxidant enzyme activity and osmotic regulation (Figure 10). It will be of interest for future research to study the nitrogen utilization mechanism in response to salinity to better understand the nitrogen effect on the salt tolerance of cotton.

## 4. Conclusions

This study showed that nitrogen supplementation was beneficial for cotton plants and helped to alleviate the toxic effects of salinity stress on the growth of two genotypes. Salt stress noticeably decreases the photosynthetic efficiency of plants by influencing ROS generation. In contrast, nutritional supplementation with nitrogen mitigated the detrimental effects of salt stress and boosted cotton-seedling growth by upregulating the activity of antioxidant enzymes and accumulated organic solutes. Additionally, this comprehensive study provided evidence that the SS genotype more effectively alleviated the salt-induced damage than the SS genotype upon nitrogen co-application in the saline media. Therefore, nitrogen application may be suggested as a vital strategy for strengthening the salt tolerance of cotton plants at the seedling stage.

## 5. Materials and Methods

### 5.1. Plant Materials, Growth Environments, and Experimental Treatments

**Figure 11:**
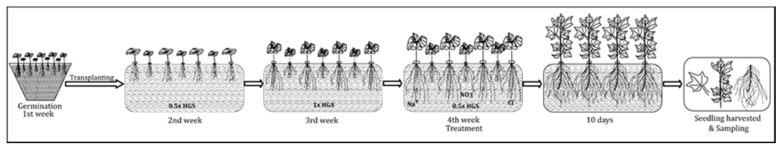
Schematic presentation of the experimental method.

The experiments were conducted in the greenhouse of the Cotton Research Institute, Chinese Academy of Agricultural Sciences, Anyang, Henan, China. The two cotton genotypes used in this study were Z9807 (salt tolerant, ST) and Z0102 (salt sensitive, SS), which were selected during an earlier screening study (data unpublished). The processes of seedling culture and experimental treatment of this study are depicted in Figure 11. Healthy and uniform seeds were surface disinfected with 2% (*v*/*v*) sodium hypochlorite solution for 15 min, followed by washing with double-distilled water four times. After disinfection, seeds were sown into a germination pot containing sterilized wet sand (10 × 10 × 10 cm). After seven days of germination, identical seedlings were transplanted to 7-L plastic boxes, covered with a black foam board, containing half-strength modified Hoagland’s nutrient solutions (12 plants per box). During the second week of growth, full-strength modified Hoagland’s nutrient solutions (2.0 mmol·L^−1^ Ca(NO_3_)_2_, 2.0 mmol·L^−1^ KCl, 0.5 mmol·L^−1^ KH_2_PO_4_, 2.0 mmol·L^−1^ MgSO_4_, 0.1 mmol·L^−1^ EDTA·Fe·Na, 46.2 µmol·L^−1^ H_3_BO_3_, 9.1 µmol·L^−1^ MnCl_2_·4H_2_O, 0.8 µmol·L^−1^ ZnSO_4_·7H_2_O, 0.3 µmol·L^−1^ CuSO_4_·5H_2_O, and 1.0 µmol·L^−1^ (NH_4_)_6_Mo_7_O_24_·4H_2_O) was supplied. The pH of the nutrient solution was attuned to 5.8–6.2, using KOH or HCl solutions. The temperature, relative humidity, and photoperiod of the growth chamber were 28 ± 2 °C, 60% ± 5%, and 16 h light/8 h dark, respectively. After two weeks of transplanting or three weeks after germination, cotton seedlings (with three true leaves) were subjected to treatments with NaCl and nitrogen. Ca(NO_3_)_2_·4H_2_O was used as nitrogen source, according to Dai et al. [28]. In total, the experiment comprised four different treatments: control (0.25 mmol·L^−1^ N without NaCl), NaCl (0.25 mmol·L^−1^ N with 200 mmol·L^−1^ NaCl), Nitrogen (2.5 mmol·L^−1^ N), and NaCl + N (200 m mol·L^−1^ NaCl and 2.5 mmol·L^−1^ N). Each treatment was conducted in three replications. To evade osmotic shock of cotton seedlings, NaCl was dissolved in the nutrient solution via a three-step process over a 3-day period. The nutrient solution and dH_2_O were replaced once per week throughout the experimental duration and the solution was constantly aerated. Moreover, the plastic boxes were frequently moved to a new position to decrease the impact of micro-environmental effects. The evaporated water was replenished every two days to re-attain the target volume. After 14 days of treatment, cotton seedlings were harvested, and the relevant parameters were analyzed.

### 5.2. Estimation of Growth Parameters

After 14 days of treatment, cotton seedlings were harvested and their shoot length (SL) was measured from cotyledon to shoot apex. The number of leaves (LN) of every single plant was counted. Six cotton seedlings were used to measure all morphological traits for each replication.

### 5.3. Biomass Determination

The treated cotton seedlings were thoroughly washed with deionized water and the water on leaf surfaces was wiped off with absorbent paper. The seedlings were divided into shoot and root parts. After that, plant materials were oven-dried at 105 °C for 30 min, followed by 80 °C for the next 24 h until a constant weight was reached. The total biomass was measured.

### 5.4. Determination of Photosynthetic Pigments

Approximately 0.05 g of fresh leaf tissue was extracted with 95% ethanol to determine the content of photosynthetic pigments (chlorophyll a and chlorophyll b). All samples were analyzed using a spectrophotometer (UV-1280, Shimadzu, Kyoto, Japan) at wavelengths of 663 and 645 nm [76]. The whole process was accomplished in a low-radiance environment.

### 5.5. Measurement of Gas Exchange Attributes

Gas exchange attributes, e.g., net photosynthetic rate (A), stomatal conductance (gsw), transpiration rate (E), and intercellular CO_2_ concentration (Ci), were measured using portable photosynthesis instruments (LI-6800, LI-COR, Inc., Lincoln, NE, USA) at the top third fully expanded leaf. The measurement was conducted on 6 cm^2^ of total leaf area. The CO_2_ concentration of the chamber was 400 µmol·mol^−1^, the airflow was constant at 700 µmol·s^−1^, and the relative humidity of the chamber was 55%.

### 5.6. Determination of Oxidative Stress Markers

The H_2_O_2_ concentration was measured by extracting 0.1 g fresh leaf samples in 0.1% TCA according to the instructions of Velikova et al. [77]. The optical density (OD) of the solution mixture was read at 390 nm.

Lipid peroxidation as malondialdehyde (MDA) was measured from the third fully expanded frozen leaf, following the procedure reported in Shi et al. [78]. The absorption of the supernatant was measured spectrophotometrically at 600, 532, and 450 nm.

Electrolyte leakage (EL) indicates damage of the cell membrane and was conducted following the protocol described by Larka et al. [79]. The EL was measured using Equation (1):

EL (%) = EC_1_/EC_2_ × 100 (1)

### 5.7. Determination of Soluble Sugar, Soluble Protein, and Free Amino Acid Contents

The soluble sugar content was determined according to the anthrone sulphuric acid method as described by Irigoyen et al. [80]. The sugar content was measured spectrophotometrically at 620 nm using glucose as standard.

According to Bradford [81], the soluble protein content was determined using the Coomassie brilliant blue G-250 reagent. Bovine serum albumin (BSA) was used as protein standard and the soluble protein content was measured spectrophotometrically at 595 nm.

To estimate the total free amino acid content, the ninhydrin reagent method was adopted according to Lee and Takahashi [82] and the absolute absorbance was read at 570 nm.

### 5.8. Determination of Leaf Relative Water Content

The leaf relative water content (LRWC) was determined according to Bars and Weatherly [83] based on Equation (2):

LRWC (%) = [(FW − DW)/(TW − DW)] × 100%(2)

where FW, DW, and TW represent the fresh weight, dry weight, and turgid weight, respectively.

### 5.9. Assay of Antioxidant Enzymes

The antioxidant enzyme assay was prepared by homogenizing 0.5 g fresh leaves in a pre-chilled mortar and pestle, using 50 mmol·L^−1^ sodium phosphate buffer (pH 7.8) containing 1% polyvinyl pyrrolidine, 0.2 mmol·L^−1^ ethylenediaminetetraacetic acid, and 10 mmol·L^−1^ magnesium chloride. The homogenate was then used as an enzyme source after centrifugation at 12,000× *g* for 25 min at 4 °C.

The activity of SOD (EC 1.15.1.1) was determined according to Giannopolitis and Ries [84]. Finally, the photo reduction of nitroblue tetrazolium chloride was recorded at 560 nm against samples incubated in the dark.

To assay CAT (EC 1.11.1.6) activity, the Havir and McHale [85] method was adopted, and the decreasing OD was recorded at 240 nm.

POD (EC 1.11.1.7) activity was assayed with guaiacol as substrate and the increased absorbance of guaiacol oxidation was measured at 470 nm [86].

### 5.10. Statistical Analysis

The data was presented as the mean value of three replicates per treatment. The statistical analysis was performed by SPSS statistics 25.0 software (SPSS Inc., USA) and treatment effects (*p* < 0.05) were analyzed by ANOVA followed by Duncan’s multiple range test.
